# TILES-2019: A longitudinal physiologic and behavioral data set of medical residents in an intensive care unit

**DOI:** 10.1038/s41597-022-01636-4

**Published:** 2022-09-01

**Authors:** Joanna C. Yau, Benjamin Girault, Tiantian Feng, Karel Mundnich, Amrutha Nadarajan, Brandon M. Booth, Emilio Ferrara, Kristina Lerman, Eric Hsieh, Shrikanth Narayanan

**Affiliations:** 1 Signal Analysis and Interpretation Lab, University of Southern California, Los Angeles, CA USA; 2 Information Sciences Institute (USC), Marina del Rey, CA USA; 3 Keck School of Medicine, University of Southern California, Los Angeles, CA USA

**Keywords:** Databases, Health occupations

## Abstract

The TILES-2019 data set consists of behavioral and physiological data gathered from 57 medical residents (i.e., trainees) working in an intensive care unit (ICU) in the United States. The data set allows for the exploration of longitudinal changes in well-being, teamwork, and job performance in a demanding environment, as residents worked in the ICU for three weeks. Residents wore a Fitbit, a Bluetooth-based proximity sensor, and an audio-feature recorder. They completed daily surveys and interviews at the beginning and end of their rotation. In addition, we collected data from environmental sensors (i.e., Internet-of-Things Bluetooth data hubs) and obtained hospital records (e.g., patient census) and residents’ job evaluations. This data set may be may be of interest to researchers interested in workplace stress, group dynamics, social support, the physical and psychological effects of witnessing patient deaths, predicting survey data from sensors, and privacy-aware and privacy-preserving machine learning. Notably, a small subset of the data was collected during the first wave of the COVID-19 pandemic.

## Abstract (summary)

**Table:** 

Measurement(s)	Stress • Burnout • Affect • Depression • Sleep • Physical Activity Measurement • Alcohol Use History • Frequency Any Tobacco Use • Personality • Social Support • Intragroup Conflict • Challenge and Hindrance Stressors • Demographics • Context and Atypical Events • Daily Stressors • Most Stressful Event • Work Context • Job Performance • Job Satisfaction • Stressors at Work • Charting at Home • Coworker Trust • Social Networks at Work • Socialization Outside of Work • Use of Wellness Resources • Heart Rate • Step Count • Acoustic Features • Team Interactions • Proximity to Key Objects • Cell Phone Use • Hospital Contextual Data • Coping with Stress • Productivity at Work • Pride at Work • Teamwork • Support System
Technology Type(s)	Perceived Stress Scale - 14 Questionnaire • Survey • Patient Health Questionnaire - 9 Item • Pittsburgh Sleep Quality Index • FitBit • International Physical Activity Questionnaire (August 2002) Short Last 7 Days Self-Administered Format • Unihertz Atom Phone • Minew E8- TILES Interaction Sensors • Minew E8- Eddystone Beach • Rescuetime • Evaluations • Patient Census • Interview
Sample Characteristic - Organism	Homo sapiens
Sample Characteristic - Location	Los Angeles County and University of Southern California Medical Center

## Background & Summary

The Intensive Care Unit (ICU), which provides care for critically ill patients, is an unpredictable and demanding environment. Physicians frequently engage in high-stakes tasks and encounter patient death and ethical issues surrounding end-of-life care^1^. The ICU may be especially challenging for physicians-in-training, as patient deaths can be traumatic for those with less experience^2^. Our first goal, therefore, was to identify how working in the ICU impacts the well-being of physicians-in-training, specifically residents (i.e., physicians who completed medical school but practice under supervision). Understanding factors that contribute to resident well-being is critical, as residents report higher levels of depression and burnout than the general population^3^. Poor well-being may also have significant consequences for patient care, as burnout is linked with lower quality care and more medical errors^4^.

Our second goal was to examine team functioning in a demanding workplace. Several studies suggest that working under extreme circumstances may lead to decreased cooperation and communication quality^5,6^. Conversely, working under extreme circumstances may create solidarity^7^. In the ICU, residents are supervised by an attending physician and a fellow (i.e., physicians who completed residency and are receiving critical care medicine training). Junior residents work closely with more senior residents and all residents coordinate care with nurses. Our study sought to shed light on how team interactions fluctuated in conjunction with workplace demands. Understanding whether demanding situations can affect team functioning is vital, as some studies have found a link between teamwork quality and patient outcomes^8^.

Most studies on individual and team functioning among physicians are cross-sectional (e.g.^6,9–11^), with a notable exception being a study that examined real-time, within-subject associations between work activities and stress^12^. A limitation of cross-sectional designs is that they assume that these factors are stable across time, when in fact, they may be dynamic. For example, work hours may fluctuate daily depending on the patient census. Intensive longitudinal designs, which can be used to model how individuals and teams respond to day-to-day changes, are thus needed.

Most studies on individual and team functioning among physicians also rely on self-report (e.g.^6,9,13,14^). Recent advances in bio-behavioral sensing enable continuous and unobtrusive measurement of heart rate and sleep quality^15^. Advances in the Internet-of-Things (IoT) and cloud computing enable interactions to be studied using off-the-shelf devices. Raw audio can also be processed in-device so that information regarding the quality of interactions can be extracted while preserving privacy. We leveraged these bio-behavioral sensing opportunities together with self-report measures.

This study extended our prior work which also used an intensive longitudinal design and a combination of bio-behavioral sensing and self-report measures to examine well-being and job performance among healthcare professionals^16^. Our current study is unique because participants worked in the same unit, enabling us to observe team-level interactions, and because participants were still in training, which may give greater insight into how individuals acclimate to a demanding workplace.

We observed 57 residents during their three-week ICU rotation at the Los Angeles County and University of Southern California Medical Center (LAC + USC). Residents completed baseline measures of mental and physical health, social support, and personality, and were interviewed. They also completed daily surveys that assessed stress, sleep, job performance and satisfaction, and weekly surveys that assessed exercise, substance use, and relationships with teammates. They wore sensors such as a Fitbit, a smartphone that captured audio features, and a Minew sensor (referred to as the TILES Interaction Sensor or TIS) that detected when they were in proximity to others wearing the sensor, as a proxy for team interactions. Uniquely, we recruited full-time employees who worked with the residents (e.g., nurses) to wear the TIS device, so that a more complete picture of residents’ interactions could be obtained. Lastly, we obtained job performance evaluations and hospital contextual data (e.g., patient census).

Our data set has applications in health psychology, industrial-occupational psychology, medical education, signal processing, and machine learning/AI. Our data set also presents opportunities to model changes due to the COVID-19 pandemic, as a small subsample of residents were participating when the first cases arrived at the hospital on March 23, 2020.

The data are available at: https://tiles-data.isi.edu/^17^.

## Methods

### Location and Setup

The study took place in the ICU of the Los Angeles County and University of Southern California Medical Center (LAC + USC). The ICU, which had a capacity of fifty beds, was divided into two units. Each team consisted of an attending physician, a Pulmonary, Critical Care, and Sleep Medicine fellow, and six to seven residents. Most residents rotated through the ICU for three weeks.

Residents’ shifts generally followed the schedule outlined in Table 1, which consisted of both individual and team management of patients. From 6:30 to 7:00 AM, residents received updates from the night team (i.e., sign out), reviewed charts, and divided up the work for the day (i.e., pre-rounds). Led by the fellow, the residents on the team then saw all the patients in the unit from 7:00 to 8:30 AM in order to develop a management plan (i.e., fellow rounds). After fellow rounds, residents continued seeing patients (i.e., work rounds) until the attending arrived at 10:00 AM. From 10:00 AM to 12:00 PM, the attending led the residents and fellow in seeing all the patients in the unit. The attending reviewed new admissions, residents’ work on patient charts, and identified plans for patient care and discharges. From noon to 1:00 PM, residents took their lunch break and on occasion, attended lectures at the hospital. After lunch, they provided care, updated patients’ charts, admitted new patients and discharged patients from the ICU. They may also attend lectures. At the end of their shift, residents briefed the night team on what occurred during the day.

**Table 1:** Day shift schedule. This table describes residents’ general schedule.

Start time	End time	Activity
6:30 AM	8:30 AM	Sign-out, Pre-rounds, and Fellow Rounds
8:30 AM	10:00 AM	Work Rounds
10:00 AM	12:00 PM	Attending Bedside Rounds
12:00 PM	1:00 PM	Lunch/Conference (Grand Rounds, M&M, CPC)
1:00 PM	5:30 PM	Patient Care and Management/Afternoon Didactics
5:30 PM	6:00 PM	Sign-out to Night Float resident

Note that schedules varied and occasionally residents began working in the ICU later or left earlier due to professional activities.

Residents will be referred to as *focal* participants here while non-resident participants, who only wore the TIS and did not complete surveys or interviews will be referred to as *minimally involved participants*.

### Materials

#### Baseline Survey

The baseline survey is comprised of the following scales:

- **Perceived Stress.** Stress in the past month was measured using the *Perceived Stress Scale* (PSS)^18^. The PSS consisted of ten items that were rated on a five-point Likert scale ranging from never (0) to very often (4). Some items were reverse coded as required and the ratings for the ten items were averaged to create a composite score (possible range: 0–4).
- **Burnout.** Burnout was measured using the *Oldenburg Burnout Inventory* (OBI)^19^. The OBI was comprised of 2 eight-item subscales: disengagement and exhaustion. Items were rated on a four-point scale ranging from strongly agree (1) to strongly disagree (4). Some items were reverse coded and the items for each subscale were averaged (possible range for each subscale: 1–4).
- **Affect.** Affect in the past week was measured using the *Positive And Negative Affect Schedule* (PANAS)^20^. The PANAS consisted of two subscales: positive and negative affect. Each contained 10 items that were rated on a five-point scale from very slightly or not at all (1) to extremely (5). The items for each subscale were summed to create an aggregate score (possible range for each subscale: 10–50).
- **Depression.** The nine-item *Patient Health Questionnaire* (PHQ-9) was used to assess depressive symptoms in the past two weeks^21^. The items were rated on a four-point scale from not at all (0) to nearly every day (3) and then aggregated (possible range: 0–27).
- **Anxiety.** The *trait* subscale (denoted STAI-T) of the *State and Trait Anxiety Inventory* (STAI) was used to assess symptoms of anxiety^22^. The trait subscale of the STAI consisted of 20 items, which were rated on a four-point scale from almost never (1) to almost always (4). Some of the items were reverse coded as requred. The items were then aggregated (possible range: 20–80).
- **Sleep.** The 12-item *Medical Outcomes Study* (MOS) *Sleep Scale* was used to measure sleep quantity and quality in the past four weeks^23,24^. Participants reported the number of hours slept and a dichotomous optimal sleep score was computed (i.e., whether participants slept between 7–8 hrs per night). The sleep quality subscales that were computed were sleep disturbance (mean of 4 items), snoring (1 item), awakening with short of breath or headache (1 item), sleep adequacy (mean of 2 items), sleep somnolence (mean of 3 items), and sleep problems (mean of 9 items). The possible range for these subscales was 0 to 100.
- **Physical Activity.** An abbreviated version of the International *Physical Activity Questionnaire* (IPAQ) was used to assess physical activity^25^. Participants reported the total amount of time engaging in vigorous and moderate activities across all domains (e.g., at work, for leisure) and the amount of time walking. Their responses were then converted into total MET-minutes, which is a measure of the intensity and duration of physical activity where higher scores indicate greater physical activity. Participants also reported the amount of time spent sitting in the past seven days.
- **Alcohol Use.** The *Habitual Alcohol Use* (HAU) scale was used^26^. Items included the number of alcoholic drinks consumed in a typical week, their drinking rate (i.e., number of drinks consumed per hour), and the tendency to get drunk while drinking. The items were weighted and summed.
- **Tobacco Use.** An abbreviated version of the *Global Adult Tobacco Use Scale* (GATS) was administered^27^. Participants reported whether they used tobacco and the total units of tobacco smoked in a typical week.
- **Personality.** The *Big Five Inventory 2* (BFI-2) was used to assess five dimensions of personality: extraversion, agreeableness, conscientiousness, negative emotionality (formerly known as neuroticism), and openness to experience^28^. Each subscale consisted of twelve items that were rated on a five-point scale from disagree strongly (1) to agree strongly (5). The mean of each subscale was then computed (possible range: 1–5).
- **Social Support.** The *Multidimensional Scale of Perceived Social Support* (MSPSS) was used to assess perceived support from romantic partners, family, and friends^29^. Each of these three subscales were comprised of four items. These items were rated on a seven-point scale from very strongly disagree (1) to very strongly agree (7). The mean of each subscale was computed (possible range: 1–7).
- **Intragroup Conflict.** The *Intragroup Conflict Scale* consisted of two subscales: conflict due to tasks, and conflict due to interpersonal relations^30^. These subscales contained four items each and were rated on a five-point scale from none (1) to a lot (5). The mean of each subscale was computed (possible range: 1–5).
- **Challenge and Hindrance Stressors.** The *Challenge and Hindrance Stressors Scale* (CHSS) was a 16-item scale consisting of two eight-item subscales: challenge stressors (i.e., job demands that create opportunities for personal growth) and hindrance stressors (i.e., job demands that hinder opportunities for growth)^31^. Items were rated on a five-point scale ranging from strongly disagree (1) to strongly agree (5) and the mean of each subscale was generated (possible range: 1–5).
- **Demographics.** Participants reported their gender, relationship status, number of children in household, housing situation (e.g., renting a private apartment, townhouse, condo, or house), people with whom they lived, mode of transportation to work, commute time, and household income in 2019. Household income was dichotomized as $74,000 or below, which is the range in which participants’ salaries fall, or $75,000 and above. Participants’ year in training and the unit in which they worked were obtained from the residency programs. Age, ethnicity, country of birth, and whether English is their native language, have been excluded from the shared data set so that participants cannot be easily re-identified.

#### Ecological Momentary Assessment

Participants received two surveys daily (including non-work days): a one-item survey on current stress levels in the middle of the workday (hereafter named the *midday survey*) and a survey on daily stressors, work behaviors, and sleep at the end of the workday (hereafter named the *end of day survey*). Participants received the midday survey at 12:15 pm during their lunch break and the end of day survey at 6:15 pm. The midday survey stayed open for four hours and the end of day survey stayed open for six hours.

At the end of the week, participants received an end of week survey (hereafter named the *end of week survey*) in addition to the end of day survey. The end of week survey contained questions on substance use, exercise, work stressors, relationships with co-workers, and the utilization of wellness resources offered by the school of medicine and was administered in conjunction with the end of day survey. These items were only asked weekly in order to minimize participant burden and maximize response rates. As participants’ ICU rotations began on a Tuesday, participants received the end of week survey on Mondays.

- **Midday Survey.** Participants indicated how stressed they were that morning on a scale from not at all stressed (1) to a great deal of stress (7).
- **End of Day Survey**
  - **Context and Atypical Events.** Participants indicated what they were doing (e.g., work, leisure, household activities) and where they were (e.g., home, work, outdoors) immediately before taking the survey. They also listed any atypical events that had occurred or were expected to occur that day.
  - **Stress.** Participants indicated how stressed they were that afternoon on a scale from not at all stressed (1) to a great deal of stress (7) (same as the midday survey).
  - **Daily Stressors.** Participants indicated whether they had experienced stress that day from any of the following: Tension or arguments with spouse/partner; tension or arguments with family members; an item breaking; not having enough money to pay bills, loans, or something else that is needed; finding time for self-care; own health problems; someone else’s health problems; doing or needing to do household tasks; caring or arranging care for child; and experiencing discrimination. Participant selected all that applied.
  - **Most Stressful Event.** Participants were asked to report the most stressful event that occurred that day and the time at which it occurred (e.g., morning, midday, afternoon). Participants’ responses were coded and to protect participants’ privacy, the codes are presented in the data set, rather than the open-ended responses. Codes include patient coded (e.g., when the participant experienced cardiac arrest), many patients, goals of care/family discussions, and ran late/tried to be on time.
  - **Work Context.** Participants indicated whether they participated in half-day non-clinical activities (e.g., didactics), and therefore were not in the ICU, or were out sick. They also indicated when they started and ended work.
  - **Job Performance.** Perceived job performance was a single-item scale (i.e., “How would you rate your overall work performance today?”). Participants responded on a six-point scale ranging from poor (1) to excellent (6). This was followed by an open-ended question on the aspect of work that participants felt they had performed the best that day. Again, responses were coded in order to protect sensitive information. Codes include provided good care/managed patients well, rapport with patient/compassionate care, end of life/goals of care, and helped team.
  - **Job Satisfaction.** Job satisfaction was also a single-item scale (i.e., “Select the face that best describes how you feel about your job today.”). Participants selected from one of five emojis^32^.
  - **Sleep.** Two items from the *Pittsburgh Sleep Quality Index* (PSQI) were used^33^. Specifically, participants reported the number of hours that they slept last night and rated the overall quality of their sleep on a four-point scale ranging from very bad (1) to very good (4).
- **End of Week Survey.** The following questions were included on the end of week survey:
  - **Alcohol Use.** Participants indicated whether they consumed alcohol that week and the number of beverages they consumed.
  - **Tobacco Use.** Participants indicated whether they used tobacco products that week and the number of tobacco products they used.
  - **Physical Activity.** Participants indicated whether they exercised that week and the number of hours they spent exercising. The intensity of the activity was not documented.
  - **Stressors at Work.** Participants indicated the degree to which they felt stressed by the following work events: difficulty finding a computer, difficult patient interactions, conflict with co-workers, too many patients to care for, had to stay late to care for patients, was paged into work to provide emergency medical care, had to supervise more junior residents or staff, clinical and administrative responsibilities, difficult patient cases, and patient death. These items were developed by the research team following an exploratory focus group interview with six residents prior to the full study. The scale ranged from not at all stressful (1) to very stressful (7) and a “not applicable” option was also available. These items were not summed or averaged.
  - **Charting at Home.** Participants were asked whether they brought work-related tasks home. Specifically, they indicated whether they spent time charting at home (i.e., documenting patient care) that week and the number of hours spent doing so.
  - **Coworker Trust.** Coworker trust was measured using four items that were rated on a five-point scale ranging from strongly disagree (1) to strongly agree (5)^34^. The mean of these items was computed.
  - **Social Networks at Work.** Participants listed the names of up to three team members for the following four categories:1) team members with whom they asked work-related questions in-person, 2) team members with whom they asked work-related questions digitally, 3) team members with whom they shared positive (joys, successes) or negative (frustrations, challenges) experiences from work in-person, and 4) team members with whom they shared positive or negative experiences from work digitally.
  - **Socialization Outside of Work.** Participants indicated whether they attended social events organized by the residency program and whether they spent time with other residents informally that week.
  - **Use of Wellness Resources.** Participants indicated whether they utilized the school of medicine’s wellness resources that week.

#### Sensing Devices

Sensing devices consisted of wearable sensors and environmental sensors (see Table 2). Wearable sensors were given to focal participants. Minimally involved participants received a passive version of the *TILES Interaction Sensor* (TIS) described below. Environmental sensors were used for the localization of participants within the ICU.

- **Fitbit Charge 3 (Wearable).** Participants were given this smart wristband with instructions to wear it continuously during the study and to recharge it while showering. Collected data included heart rate, step count, and sleep quantity and quality.
- **Unihertz Atom Phone (Wearable).** Focal participants wore this small Android smartphone at work by clipping it to their clothing near the neckline or by placing it in their shirt pockets. The Atom phone ran custom software and served as a social-sensing, environment-sensing badge, and audio collection device using Bluetooth and its built-in microphone. The audio collection capability was handled by the *TILES Audio Recorder* (TAR), which immediately processed recorded audio samples by storing anonymized features and discarding the recording^35^. Participants charged their phones when they were not at work.
- **Minew E8 - TILES Interaction Sensor (TIS) Device (Wearable).** This Minew E8 was programmed with one of two possible custom-designed firmwares: active mode and passive mode. Its compact size (36.5 × 23.7 × 5 mm) enabled it to be temporarily affixed on the participants’ work badge. Both firmwares acted as beacons, regularly broadcasting custom designed Bluetooth advertisement packets (to be received by active Atom phones, active TIS sensors and Owl-in-Ones). Focal participants wore the active TIS variant which, on top of acting as beacons, also recorded *Received Signal Strength Indicators* (RSSI) from nearby Eddystone beacons and other TIS devices. The passive TIS variant was given to minimally involved participants.
- **Minew E8 - Eddystone Beacon (Environmental).** These Bluetooth implemented the Eddystone BLE advertisement protocol and were attached to key objects in the ICU (e.g., computer monitors, patient beds) to facilitate proximity-based detection of focal and minimally involved participants.
- **reelyActive Owl-in-One (Environmental).** These Bluetooth hubs were positioned within the ICU, the resident lounge, and the conference rooms. They collected Bluetooth information from Eddystone beacons and TIS devices described above and from other Owl-in-One hubs.

**Table 2:** Selected sensors with a summary of measures (output) and instructed use or sensing times.

Sensor	Measures	Instructed use/Sensing times
Fitbit Charge 3	PPG-based heart rate, step count, sleep	24 h/day
RescueTime	Cell phone usage	24 h/day
Unihertz Atom	Audio features, received signal strength of Bluetooth packets	At work
TILES app	Midday, end of day, end of week surveys	Upon request (push event)
Web browser	Baseline survey	At the beginning of the study
Owl-in-One	Received signal strength of Bluetooth packets	Installed at LAC + USC Hospital, 24 h/day
Eddystone Beacon	Eddystone Beacon	Installed at LAC + USC Hospital, 24 h/day
TIS Device	Interactions	At work

The first two sensing streams were obtained directly from participants through wearable sensors while apps were installed on personal smartphones. All surveys were obtained by direct input of participants on their personal smartphones or a web browser. Owl-in-One and Eddystone beacons sensing streams were obtained by placing sensors in key locations of the ICU. The TIS device streams were obtained by placing a contact tracing sensor on the participants’ work badge. PPG: photoplethysmography.

#### Phone Apps

We used three smartphone applications

- **TILES app.** The TILES application, which was a modified version of the application used in our previous study^16^, was used for consenting participants, administering the surveys, and retrieving Fitbit data. It was installed on participants’ personal smartphone (iOS or Android).
- **RescueTime.** This third party application was installed on participants’ personal smartphone and was used to monitor cell phone usage.
- **TILES Audio Recorder (TAR).** The TILES Audio Recorder (TAR) was installed on the Atom phone and was used for audio and Bluetooth proximity recording and processing. It collected fewer features than the TAR used in our previous study^16^ but it still captured the same amount of information from audio modality.

#### Interviews

Interviews were used to obtain rich descriptions of participants’ experiences working in the ICU and their sources of support. Participants were interviewed at the beginning of their rotation (i.e., pre-study interview) and after their rotation ended (i.e., post-study interview). For the pre-study interview, participants were asked about their work experiences and sources of support in the month prior to their ICU rotation. For the post-study interview, participants responded to these questions in the context of their ICU rotation. Conducting interviews at the beginning, as well as, the end of the rotation, allowed for greater insights into the experiences that are unique to working in the ICU. We asked:

1. *How stressed do you feel this week in comparison to last week? Why do you feel more stressed/less stressed/about the same?*
2. *Can you give me an example of how you dealt with a stressful situation at work in the last month/in the ICU?*
3. *Can you describe a time when you felt productive or efficient at work in the last month/in the ICU?*
4. *Can you describe a time when you felt proud at work in the last month/in the ICU?*
5. *What did you enjoy about working with your team in the last month/in the ICU? What did you find challenging about working with your team in the last month/in the ICU?*
6. *Can you name people (maximum of five) who were in your support network during the last month/during your ICU rotation (you can name your relationship to them, rather than their name). How did they support you?*

#### Job Performance Evaluations

At the end of every rotation, job performance evaluations are given by the attending(s) that supervised them and their teammates. Over the course of residency, the job performance evaluations are tracked and assessed to ensure that residents hit the necessary training milestones to become eligible for initial certification and are ready for independent practice. The residency directors review the evaluations on a monthly basis.

These evaluations that were used in this study were designed by the residency program and based on the developmental milestones set by the American Board of Internal Medicine. Participants were given the option on the consent form to decline having employee data, such as job evaluations, accessed by researchers.The anonymized evaluations are available in the data set as a measure of job performance. Refer to Table 3 for the items.

- **Attending Evaluations.** Forty-seven participants granted the research team permission to access their job evaluations. The number of evaluations participants received varied, as not all of the attendings completed their evaluations. Moreover, attendings rotated through different services and their schedules were not aligned with residents; thus, some participants were evaluated by multiple attendings. Nonetheless, all but one participant received at least one evaluation (43% 1 evaluation, 47% 2 evaluations, 9% 3 evaluations). Participants were rated on their patient care and procedural skills, medical knowledge, system-based practices, practice-based learning and improvement, professionalism, and interpersonal and communication skills on a scale from 1 to 5 (1.0 = at the level of an intern (i.e., first-year resident), 1.5 = between intern and PGY2 (i.e., postgraduate year 2, second-year resident), 2.0 = at the level of a PGY2, 3.0 = between PGY2 and PGY3 (i.e., postgraduate year 3, third-year resident), 3.5 = at the level of PGY3, 4.0 = above the level of a PGY3, 5.0 = aspirational).
- **Peer Evaluations.** Peer evaluations were optional; thus, only half of participants (n = 23) received a peer evaluation. Fifteen percent of participants received 1–3 evaluations, 28% received 4–6 evaluations, and 6% received 7–9 evaluations. Participants were rated on their interpersonal and communication skills, patient and procedural skills, and professionalism on a scale from 1 to 5 (1 = unsatisfactory, 2 = marginal, 3 = average, 4 = good, 5 = outstanding).

**Table 3:** Attending and peer evaluation items.

Evaluator	Item	Description
Attending	Patient Care and Procedural Skills	Gathers and synthesizes essential and accurate information to define each patient’s clinical problems and to develop comprehensive management plan for each patient
	Medical Knowledge	Clinical knowledge: knowledge of diagnostic tests and procedures
	System-Based Practices	Works effectively with an inter-professional team (e.g., peers, consultants, nursing, ancillary professionals and other support personnel) and recognizes system error to advocate for system improvement
	Practice-Based Learning and Improvement	Receptive to constructive criticism
	Professionalism	Accepts responsibility and follows through on tasks
	Interpersonal and Communication Skills	Has professional interactions and communicates effectively with patients, caregivers, and members of the inter-professional teams
Peer	Interpersonal and Communication Skills 1	Effective communication with families, patients, and all members of the health care team (nursing, peers, faculty, and other services)
	Interpersonal and Communication Skills 2	Teaching/willingness, ability, provides opportunities to learn/teach
	Interpersonal and Communication Skills 3	Supervision for patient care, during procedures, during work rounds
	Patient Care and Procedural Skills	Effectiveness and completeness of sign-outs. Information is correct and “call” plan is clearly identified
	Professionalism 1	Helpfulness in the completion of tasks and shares equally in the team’s work
	Professionalism 2	Coverage of cross-cover issues and completion of necessary tasks when on call
	Professionalism 3	Level of integrity, honesty, citizenship, trustworthiness, and reliability

#### Hospital contextual data

We obtained patient census and death records from the hospital to contextualize participants’ daily work demands.

- **Patient Census.** A list of patients that were in the ICU during the study period was obtained. For each patient, we received the date and time when they were admitted to the ICU, the unit they were in, and the date and time they were discharged from the ICU.
- **Patient Deaths.** A list of deaths that occurred during the study period was also obtained (no names were given). For each patient death, we received the corresponding date, time, unit, and room number.

### Study Procedures

All these steps were conducted in accordance with University of Southern California’s Institutional Review Board (IRB) approval (study ID HS-19-00606).

#### Requirements for Eligibility

Residents were invited to participate as *focal participants* if they rotated through the ICU between November 2019 and April 2020. In addition, they needed to be proficient in speaking and reading English and have access to an Internet and Bluetooth-enabled personal smart phone (not used by anyone else) running Android 4.3 or iOS 8 or newer, a personal email account, and WiFi at home.

Full-time employees who worked with the residents in the ICU were invited to participate as *minimally involved participants*. These included attending physicians, fellows, nurses, and a small number of residents whose rotation only partially overlapped with the study.

#### Recruitment

Residents who were rotating through the ICU between November 2019 and April 2020 were contacted by their program coordinators. Those who expressed interest were asked to complete a screening survey to determine their eligibility. Eligible residents were then scheduled for an enrollment session. They also received a text with the link and directions to download the TILES study app.

Research team members approached minimally involved participants at the start or end of their shifts to inform them about the study. Minimally involved participants were also informed about the study through email and flyers posted in the ICU.

#### Participant Enrollment Session

Residents who indicated their interest were invited to a one-hour enrollment session prior to the start of their rotation. However, due to scheduling conflicts, 11 participants (19%) were enrolled on their first day of their ICU rotation. During the enrollment session, residents who had not done so downloaded the TILES study app and indicated their consent via the app. They received their sensors (i.e., a TIS device, a Fitbit, and an Atom phone) and instructions on when and how to wear them. They were shown how to sync their Fitbit and Rescuetime accounts and received information on the survey and interview schedules and the environmental sensors that were placed in the ICU. They were also informed about the compensation scheme and were given their gift card, which was loaded weekly.

Minimally involved participants who consented to participate were shown how to wear their TIS devices and were informed about their compensation.

#### Baseline Survey

After the enrollment session, participants received a link to the baseline survey. They were instructed to complete the survey prior to the start of data collection or for those who were enrolled on the first day of their ICU rotation, as soon as possible. The survey was administered through Qualtrics and could be completed on a computer or mobile device. The median time to complete the survey was 31 minutes (Q1-Q3 = 18 minutes-98 minutes).

#### Data Collection

**Fig. 1:**
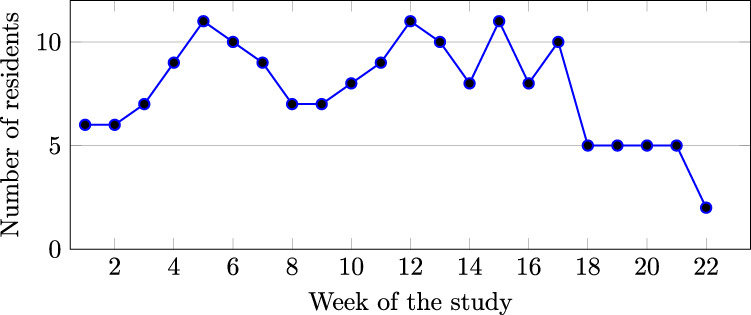
Number of residents enrolled over weeks of the study.

All residents were invited to participate in the study for four weeks, as some residents (n = 7) were scheduled to work in the ICU for four weeks. The remaining residents worked in the clinics, other units in the hospital, or were on the night shift in the ICU during the fourth week. For consistency across participants, we have made data from participants’ ICU rotation (day shift only) available. For participants who were scheduled to be in the ICU for four weeks, data from the first three weeks is available. Figure 1 shows the number of focal participants over the weeks of the study.

#### Interviews

Participants were scheduled to be interviewed by the graduate medical wellness clinician during the first two weeks of their data collection period (pre-study interview) and again after they completed data collection (post-study interview). However, due to scheduling difficulties, a subset of participants did not complete the pre-study interview (n = 8) and more than a third of participants completed their pre-study interviews after the second week (n = 24). All participants completed the post-study interview. Interviews were 15–20 minutes and detailed notes were taken by the wellness clinician. Interviews conducted on or after March 18, 2020 were conducted over the phone due to social distancing guidelines.

#### Job Performance Evaluations and Hospital Contextual Data

Job performance evaluations were obtained from the residency programs and patient census and death data were obtained from the hospital.

#### Off-boarding Session

At the end of the data collection period, participants were off-boarded by a research assistant. The off-boarding sessions lasted ten minutes and participants returned their Atom phones and TIS devices. Research assistants also confirmed that participants’ Rescuetime accounts were synced to the TILES app.

Off-boarding sessions for the minimally involved participants were also faciliated by a research assistant and consisted of participants returning their TIS devices and receiving their gift cards.

### Incentives Structure

Focal participants were compensated with a gift card and were allowed to keep the Fitbit. To encourage engagement in the study, participants were paid weekly and the amount they received was commensurate with the number of tasks they completed (see Table 5). Participants also earned points each day, which were then translated into monetary amounts (see Table 4). Depending on the number of points they accrued, participants earned up to $25 at the end of the week (see Table 5). At the end of the study, the number of points that participants earned were summed and participants were awarded bonus points for downloading and installing the TILES app, for authorizing Fitbit access, for weeks where they earned at least 205 points, and for weeks where they earned more points than they did the previous week. The top three-point earners also received bonuses of $150, $100, and $75 respectively.

**Table 4:** Incentives structure: Points.

Kind	Action	Points
Enrollment	Download and install the TILES app	50
	Authorize Fitbit access	50
Weekly Base	Open the TILES app	5
	Complete end of day/week survey	8
	Complete midday survey	2
	Wear and sync Fitbit	10
Weekly Bonuses	Multiplier for 3 + consecutive days of Fitbit data	× 2
	Reach at least 205 weekly points	20
	Earn more points than the previous week	20
	Wear and sync Atom phone for at least 2 days	20

Participants received weekly points by wearing sensors and answering surveys. These were converted to weekly monetary rewards and end-of-study bonuses  (see Table 5 for the prizes).

**Table 5:** Incentives structure: Compensation.

Kind	Minimum # points/Rank	Prize
Enrollment	Enrollment session	$50
	Baseline survey	$50
	Offboarding session	$15
Weekly	200	$25
	150	$20
	100	$15
	75	$10
Ranking	1st	$150
	2nd	$100
	3rd	$75

Weekly points awarded for compliance were translated into monetary rewards.

**Table 6:** Internal consistency for the baseline survey.

Scale	Internal Consistency	α Baseline
Perceived Stress (PSS)^18^	0.84 ≤ α ≤ 0.86	0.88
Burnout (OBI)^19^		
Disengagement	0.79	0.81
Exhaustion	0.74	0.75
Affect (PANAS)^20^		
Positive Affect	0.86 ≤ α ≤ 0.90	0.92
Negative Affect	0.84 ≤ α ≤ 0.87	0.85
Depression (PHQ-9)^21^	0.86 ≤ α ≤ 0.89	0.86
Trait Anxiety (STAI-T)^22^	0.86 ≤ α ≤ 0.95	0.93
Sleep (MOS)^51^		
Sleep Disturbance	0.80 ≤ α ≤ 0.82	0.69
Sleep Adequacy	0.76 ≤ α ≤ 0.82	0.82
Sleep Somnolence	0.63 ≤ α ≤ 0.73	0.53
Sleep Problems (9-item version)	0.78 ≤ α ≤ 0.83	0.71
Personality (BFI-2)^28^		
Extraversion	0.88	0.90
Agreeableness	0.83 ≤ α ≤ 0.85	0.73
Conscientiousness	0.86 ≤ α ≤ 0.88	0.84
Negative Emotionality	0.90	0.92
Open-Mindedness	0.84 ≤ α ≤ 0.85	0.74
Social Support (MSPSS)^29^		
Family	0.87	0.86
Friends	0.85	0.90
Significant Other	0.91	0.98
Intragroup Conflict^30^		
Relationship Conflict	0.92	0.91
Task Conflict	0.87	0.92
Challenge and Hindrance Stressors^31^		
Challenge	0.92	0.90
Hindrance	0.83	0.80

Internal consistency was determined using cronbach’s *α* and was not applicable for the IPAQ scale, HAU scale, GATS, and single-item scales on the MOS.

Minimally involved participants were compensated with a $125 gift card.

### Data Acquisition and Flow

**Fig. 2:**
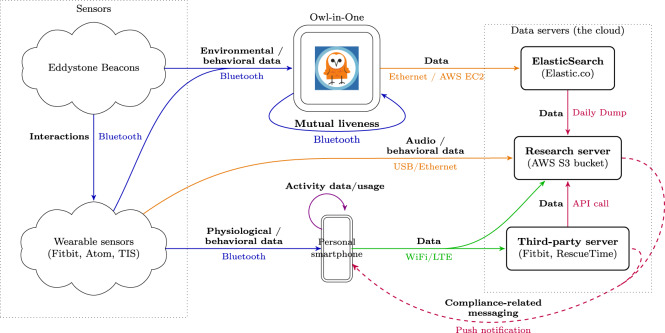
Data flow. This diagram shows the data flow from the wearable and environmental sensors to the research server. The *research server* denotes a set of AWS services that we collectively call the research server. Data is processed through these services before being stored in AWS S3 for mid-term use.

Figure 2 depicts the architecture for the data collection from sensors where information flows from left to right. The left column contains the wearable and environmental sensors providing the data (except for Owl-in-Ones, which serve a double purpose of sensing and processing/sending data). The Fitbit wristband connected to participants’ personal smartphones using Bluetooth. The data was uploaded to a third-party server using an available wireless internet connection (WiFi or LTE) through participants’ smartphones.

The Atom phones stored the data in the device, and data was uploaded manually using the computer through a USB connection. Acquired data included anonymized audio features derived from vocal audio recordings collected by the phone’s internal microphone, and timestamped proximity data obtained throughout the study by detecting Bluetooth pings from broadcasting devices (Eddystone beacons, TIS devices, and Owl-in-Ones) with timestamps.

The Owl-in-Ones received data from the Eddystone beacons and TIS devices. Data was sent through LAC + USC Internet connection (ethernet) using User Datagram Protocol (UDP) to an Amazon Elastic Compute Cloud (AWS EC2) instance which performed filtering against a white list of devices in the study and forwarded (through a RESTful API) the data to an ElasticSearch cluster provided by Elastic.co. Daily dumps of this data were stored securely in the research server.

Data was also collected directly through the participants’ personal smartphones through the TILES app, the RescueTime app, and the Fitbit servers. The TILES app uploaded data directly to the research server while the RescueTime app uploaded data to the RescueTime server and this data was later pulled by the research server. The research server^36^ included a RESTful API hosting a series of endpoints to collect push-type data streams (i.e., TILES app) in addition to a suite of tasks to fetch pull-type data streams (i.e., Fitbit, RescueTime).

### Data Preprocessing

#### Survey Data

The surveys were scored using SPSS^37^. In a few rare cases, participants completed the baseline survey more than once. In those instances, the most complete response that was submitted before or during the first week of data collection was kept. One participant submitted their response after the first week of data collection. Their response was excluded, as the data would no longer represent how the participant felt at the beginning of the rotation. In three of the cases, participants had submitted a response previously; thus, baseline data was only missing for one of the participants.

#### Fitbit Data

Preprocessing of the Fitbit data was very close to that of our previous study^16^. Fitbit data retrieved using the Fitbit API contained separate time series for measured heart rate and step count, in addition to a daily summary of physical activity and sleep. Heart rate data was reported on non-uniform intervals anywhere between approximately 5 s and 15 min depending on the participants’ physical activity. Occasionally, long strings of repeated identical heart rate values (usually 70bpm) were reported in the raw data. These strings were typically under 15 minutes but a few were as long as 20 hours. These strings were interpreted as artifacts because of consumer observations that Fitbit technology sometimes incorrectly reported exactly 70 bpm^38^ and because repeated measures of the same heart rate over several minutes is highly unlikely. Thus, sequences of at least 50 repeated identical heart rate values were replaced with NaN (Not a Number, equivalent to missing values). The step count, daily summary, and sleep data did not contain these long string artifacts and, therefore, were not pre-processed.

Finally, as the Fitbit stores local time only and does not record when time offset changes occurred, we included Fibit sync data with the Fitbit server to indicate the time offset (namely the time offset of the participant’s phone and fitbit sync times). This is a known limitation of Fitbit devices.

#### Owl-in-One Data

Bluetooth packets were binary encoded as *raddecs*^39^ by the Owl-in-Ones, transmitted over the network, and decoded into a JSON structure. Owl-in-ones aggregated Bluetooth packets received from individual devices over a fixed time window. Each raddec contains (i) the timestamp of the first data received from the device, (ii) the number of received packets (called the *number of decoding*), (iii) the average RSSI, (iv) the MAC addresses of the Bluetooth device and the Owl-in-One, and (v) the received packets. We compiled this data based on the unique identifier from each device.

#### Atom Phone Proximity Data

The Atom audio badge sensor captured packets from all Bluetooth devices broadcasting Bluetooth advertisements installed at the study location. The application running on the Atom audio badge filtered the Bluetooth packets received against a white list and kept the packets from the Eddystone beacons, TIS devices, and Owl-in-Ones. The RSSI information was pre-processed separately for the Eddystone beacons, TIS devices, and Owl-in-Ones, and stored locally. The MAC addresses associated with TIS sensors were translated into the unique participant identifiers.

The MAC addresses received alongside Bluetooth transmissions from Owl-in-One and Eddystone beacons were mapped to hospital rooms or locations and formatted into a directory name. We hashed the actual directory names, such as the floor number, unit, and room numbers, to keep the hospital’s floor plans private^16^. We used the following format: 

[building name]:[floor#]:[wing/area]:[room type][room #].

#### Atom Phone Audio Data

Features were extracted on device using a suite of 69 low-level descriptors that are a combination of filterbank data and prosodic features. The Atom phones ran the OpenSMILE toolkit^40^ to extract features from 25 ms windows every 10 ms. Custom feature configuration file was created specifically for this data collection^41^. Given the sensitive nature of the data, the computed features were stored on device and the raw audio collected was discarded. To be mindful of participant privacy, only prosodic features were included in this data set release. We plan to release the filterbank features in the future after we ensure the speech content is obfuscated using privacy preservation methods.

For data cleanup and pre-processing, wWe removed all empty files and removed duplicated columns that occur in some files. For each﻿ ﻿a﻿udio sample containing non-empty feature data, the likelihood that audio sample was produced by the participant carrying the Atom device was estimated, which we called the “foreground audio” probability. We reused the approach outlined in our previous study^16^ to produce this estimate, with a small modification to the dimension of several CNN layers in the VGG-slim^42^ neural network to accommodate the larger number of mel-frequency cepstral coefficients (MFCCs) collected in this study (40 opposed to 13). The foreground audio probability estimate ranged from 0 (highly unlikely) to 1 (highly likely).

#### Interviews

The interviews were coded using descriptive codes. Discrepancies in the coding were identified and discussed until agreement was reached. These codes (e.g., teammates were fun, teammates were supportive) were then coded into broader themes (e.g., positive environment). Both the initial codes and the themes were provided in the data set. The original responses were not provided in order to protect participants’ privacy.

#### Job Performance Evaluations

As some participants were evaluated by multiple attendings or peers, the mean score for each item (e.g., attending evaluation- medical knowledge, peer evaluation- professionalism item 1) was computed.

#### Hospital Contextual Data

We created two versions of the census data that differ in their granularity. Data users may select the version that best suits their research questions. In the first version, the data were divided into 1-hour time windows. For each of the two units in the ICU, we calculated the following: 1) the number of admissions in each time window, 2) the number of discharges during the time window, 3) the number of patients at the end of the time window, and 4) the maximum number of patients during the time window. In the second version, the data was segmented into 30-minute time windows. The names of the units were anonymized in both versions.

## Limitations

One limitation was that we were not able to deploy a follow-up survey due to participants’ limited availability. The onset of the COVID-19 pandemic also prevented longer-term follow up. Nevertheless, the post-study interview at the end of the rotation offered the opportunity for participants to reflect on their experiences as a whole after their rotation ended. A second limitation is that the social interaction data is not complete as some residents, attendings, fellows, and nurses chose not to participate. Participation rates, however, were still high: 70% of residents, 80% of attendings, and 90% of fellows.

## Data Records

The TILES-2019 dataset is hosted by the USC Information Sciences Institute^17^. See Table 7 for a summary of the data folders.

**Table 7:** TILES-2019 Main Data Record.

Folders	Subfolders	Subsubfolders	Description	File Split
fitbit	daily-summary		Daily summary (aggregates, sleep)	Per participant
	heart-rate		Heart rate (PPG)	Per participant
	sleep-data		Sleep stages time series	Per participant
	sleep-metadata		Sleep periods metadata	Per participant
	step-count		Step count	Per participant
metadata	participant-info		IDs, work information	Single file
atomproximity	tis	tis	RSSIs to each participant with TIS sensor	Single file per participant
		rssi		
	eddystone	locations	Locations within the units RSSIs to each Atom	Single file per participant
		rssi		
	owls	locations	Locations within the units RSSIs between Atoms	Single file per participant
		rssi		
owl-in-one		locations	Locations within the hospital RSSIs between Owl-in-Ones	Per participant
		rssi		
rescuetime			Personal phone usage (screen on times)	Per participant
surveys	raw	baseline	Demographics	Single file
		EMA	De-identified context questions	Single file
	scored	baseline	Scored answers	Single file
		EMA	Scored answers	Single file

There are five main folders containing information for each stream of data plus a sixth folder containing participant metadata (all presented in alphabetical order). The details of each data stream (including measurements and features) are included in each of the subfolders of the data record as README files.

### TILES-2019 Main Data Record

#### Participant Summary

We targeted for recruitment all the residents who were rotating through the ICU during the study period (i.e., between November 2019 and April 2020) and 57 (70%) agreed to participate.There were no differences in program (*χ*^2^(2, *N* = 82) = 1.87, *p* = 0.393) or in years of experience (Fisher’s exact, *p* = 0.564) between residents who participated and those who did not. Differences in demographics between residents who participated and those who did not cannot be assessed, as demographic data was not collected for residents who did not participate.

60% of the participants were male (n = 34). Most participants identified as Asian American (n = 32, 56%) or White (n = 16, 28%). A smaller percentage of participants identified as Latino/a (n = 3, 5%), African American (n = 1, 2%), multi-ethnic (n = 1, 2%), other (n = 1, 2%), or preferred not to answer/did not answer n = 3, 5%). Participants were primarily in the Internal Medicine program (n = 45, 79%) and the remaining residents were in the Emergency Medicine (n = 10, 18%) and Internal Medicine-Pediatrics (n = 2, 3%) programs. We did not include participants’ program in this data set in order to prevent re-identification. Our sample was evenly split across years of experience; there were 19 residents in their first year of training (33%), 22 residents in their second year of training (39%), and 16 residents in their third year of training (28%). Participants were given the option to decline providing audio data. Forty-five participants (79%) consented to providing audio data and 12 participants (21%) declined.

The majority of minimally involved participants who were rotating through the ICU during the study agreed to participate (80% of attendings, 92% of fellows). We did not collect data on minimally involved participants, so differences between those who participated and those who did not cannot be determined.

#### Surveys (surveys folder)

This folder contains the baseline survey file and two EMA files. The “daily-ema.csv.gz” file contains surveys responses from midday surveys and end of day surveys, and “weekly-ema.csv.gz” includes information of weekly survey answers. On the baseline survey file, each row represents the responses from one participant. The EMA file is in tall array format so that each row represents the responses from one participant at a particular timepoint. Missing responses were left blank.

#### Fitbit (fitbit folder)

We followed the same folder structure for the Fitbit data as in TILES-2018^16^.

##### daily-summary folder

Each file contains rows with a date and time and a set of daily summaries including resting heart rate, total calories burned, total number of steps, sleep report, and heart rate zone durations. The sleep reports provide information about sleep duration, sleep efficiency, the duration of four sleep stages (awake sleep, light sleep, deep sleep, REM sleep), as well as the timestamp of the start and end of the sleep. There are up to four sleep records per day. Moreover, calorie consumption and duration of four heart rate zones are available in Fitbit daily summaries.

##### heart-rate folder

Each file has rows with a timestamp and PPG-based heart rate values (beats per minute). The heart rate samples made available by the Fitbit Charge 3 sensors were aggregated over intervals of less than 1, but the time differences between two consecutive samples were non-uniform.

##### sleep-data folder

Each file has rows containing a timestamp, the sleep phase with its total duration (in), and a sleepId corresponding to a sleep metadata entry in the sleep-metadata folder. Sleep phase is either in the sleep states set (one of asleep, restless, or awake) or in the sleep stages set (one of deep, light, rem, or wake). The timestamp marks the beginning of the sleeping phase.

##### sleep-metadata folder

Each file has rows for each period of sleep, and metadata for that sleep, including beginning and end, a sleep type (main vs. nap), type of inferred sleep phases (sleep states or sleep stages), duration, and various metrics.

##### step-count folder

Each file has rows with a timestamp and step count value. In contrast to heart rate values, step count data were sampled with an interval of 1; thus, we report the number of steps taken each minute.

#### Interviews (interviews folder)

The file “p2-preinterviews-data-public-5.21.csv.gz” contains the coded pre-study interviews, and the file “p2-postinterviews-data-public-5.21.csv.gz” contains the coded post-study interviews. A value of “1” indicates that a code was applied to a participant’s interview while a value of “0” indicates that the code was not applied. The descriptions of the codes can be found in the codebook: “p2-interviews-codebook-public-5.21.csv.gz”

#### Hospital Contextual Data (hospital-census folder)

In the file “p2-census-1 hr-public-7.2021.csv.gz,” the data are segmented into 1-hour time windows. In the file, “p2-census-30 min-public-7.2021.csv.gz”, the data are segmented into 30-minute time windows. Each line corresponds to the data from each unit at a particular time window.

#### Job Performance Evaluations (job-performance folder)

The file “p2-evals-data-public-4.2.22.csv.gz” contains the attending and peer evaluations of each participant. Each row represents the evaluation scores for each participant. The file “p2-evals-codebook-public-5.21.csv.gz” contains the codebook.

#### Metadata (metadata folder)

This folder contains a file with hash-based residents participant IDs, working unit(s) (if available, using the same hashing as for the atom-proximity directories), and year in the program (1st, 2nd or 3rd year). A file indicating the dates when each participant was in the ICU is also available. Most participants (n = 53) were working in the ICU for three consecutive weeks and the exceptions are noted in the document. The schedule document also indicates the unit, which has been anonymized, in which participants were working. Furthermore, this folder includes a file with hash-based minimally involved participant IDs and their positions (N: nurse; F: fellow; A: attending).

#### Owl-in-Ones (owlinone folder)

Owl-in-One data contains information from other Owl-in-Ones (RSSIs). The Owl-in-One files are organized by Unix time days (meaning that the cutoff is midnight UTC). These files each contain the signals sent and received by the Owl-in-Ones. The sending and receiving MAC addresses and the sender and receivers’ associated directories are included.

#### Atom Proximity (atomproximity folder)

Atom-based proximity data includes information from three different sources: TIS phones (RSSIs from the TIS sensors of participants), Owl-in-Ones (RSSIs), and Eddystone beacons (RSSIs).

- tis folder. The tis subfolder is organized by participant ID. Each file contains rows with a timestamp, a participant ID of the TIS device, and the corresponding RSSI values. There are multiple files for each participant.
- eddystone folder. This folder has one file per participant. Each file contains rows with a timestamp (sorted), the hashed directory of the receiving Eddystone beacon, and the corresponding RSSI value. There is one file per participant.
- owlinone folder. The owlinone subfolder folder is organized by participant IDs. Each file includes rows with a timestamp, the hashed directory of the receiving Owl-In-One hub, and the corresponding RSSI value. There are multiple files per participant.

#### Phone timestamps (phone folder)

The files in this folder list the timestamps and the associated time zone offset. The events triggering those records are independent from the time zone offsets changes of the phone and those changes are most likely in between the events.

### TILES-2019 Audio Data Record

In this section, we describe the folder structure and contents of the TILES-2019 Audio Data Record. See Table 8.

**Table 8:** Sensor usage and compliance rates.

Sensor	Participant opt-in	Total hours	Compliance rate	Definition of compliance
Fitbit	51/57 (89%)	21,583	71%	Average fraction of days per participant with >12 hours of data
Atom	45 /57 (79%)	4,756	52%	Average fraction of work days (6 days per week) per participant with >4 hours of data
RescueTime	48/57 (84%)	—	—	Compliance cannot be estimated due to sampling scheme^†^

Compliance is computed as the presence of data exceeding half of the measurement period per day among the participants that opted in for each sensor. ^†^RescueTime data only shows the times when phone interaction occurs, thus it is not possible to differentiate between periods with no interaction and the application not working.

#### Folder Structure

##### raw-features folder

Data from each participant is organized in separate subfolders. The name of each data snippet in the participant subfolder is the unix time at which the recording started.

##### fg-predictions folder

Similarly, this folder has subfolders for each participant. This folder contains foreground audio predictions for the files in raw-features folder. These files are in NumPy (.npy) format and they store an array with the probability that the corresponding frame in raw-features folder contains foreground speech (as opposed to background speech).

#### Features

As mentioned in the Data Preprocessing section, we collected both filterbank and prosodic features, of which we are currently releasing only the prosodic features. Filterbank features consist of 40 dimensional MFCCs. The prosodic features collected are: features related to pitch/voicing like fundamental frequency, jitter, shimmer, fundamental frequency after Viterbi smoothing, energy features like: Root Mean Square energy, zero crossing rate, intensity, loudness, average energy in the spectral bands ([250Hz-650Hz], [1000Hz-4000Hz]), spectral rolloffs at 25, 50, 75 and 90 percentiles, various measures/descriptors of spectral domain like flux, centroid, entropy, variance, skewness, curtosis, slope and psychoacoustic sharpness, logarithm of harmonics to noise ratio (HNR) and harmonicity. Jitter (defined as a measure of the cycle-to-cycle variation of fundamental frequency^43^) is collected with (jitterDDP_sma) and without dithering (jitterLocal_sma) whereas shimmer (defined as a measure of the cycle-to-cycle variation of ﻿amplitude^43^) is only collected without dithering.

## Technical Validation

### Sensor Validation

#### Fitbit

There are many studies validating the data from Fitbit devices. For a list of publications, please refer to https://healthsolutions.fitbit.com/research-library/.

### TILES Audio Recorder

We presented an analysis of the audio recorder in^35^ and followed the same procedure as^16^. TAR primarily extracted the audio features using openSMILE^40^. OpenSMILE is a widely used tool for extracting a wide range of features from audio signals. To test the feature distortion from the recording device, a recording setup was proposed in^35^ to allow TAR to record speech amplified through a speaker. In this feature degradation experiment, 1000 gender-balanced utterances from the TIMIT^44^ database were randomly sampled and concatenated into one file. The audio files were then played through a loud-speaker. Multiple TARs set at distances 15 cm, 20 cm, 25 cm, and 30 cm from the speaker extracted the audio features simultaneously. This experiment quantified the feature distortion by measuring the root-mean-squared error (RMSE) and cosine distance between the features extracted from the source file and recorded features. The results showed that energy-related features were sensitive to recording distance, but pitch and spectral features yield consistent patterns with different recording distances. The results also showed that errors of pitch, MFCC, and LSP were reasonably low (e.g. pitch errors were under 10 Hz when compared with the groundtruth label), which confirmed the robustness of the feature recorded by TAR.

### Data Integrity

The compliance rates observed in this data set were similar to those found in our previous TILES-2018 data set^16^. Table 8 includes a summary of the compliance rates for Fitbit sensor, Atom sensor, and RescueTime App. Table 9 shows a measure of sensor compliance in one-week interval. The decrease in compliance in the last week of the study was not significant.

**Table 9:** Wearable sensor compliance per 1-week intervals.

Sensor	Weeks			*p*-value
	1	2	3	
**Fitbit**	72.4%	72.5%	62.0%	0.121
**Atom**	61.5%	52.8%	49.3%	0.256

The compliance rate for each participant was the percentage of days where data was available for at least half of the measurement period (Fitbit: 12 hours; Atom: 6 hours). The p-value column lists the significance test result after running a Kruskal-Wallis test of the difference in sensor compliance between weeks 1 and 3. The presented *p*-value does not suggest a significant decreasing trend in sensor compliance rate over time.

#### Sensor Data

##### Fitbit

Table 10 shows the number of Fitbit recordings across all participants. The recordings were divided by length (i.e., under 4 hours, 4–8 hours, or over 8 hours). Approximately 82.5% of the recordings were over 8 hours while close to 16% of the recordings were under 4 hours.

**Table 10:** Fitbit and Atom sensor usage.

Sensor	Usage	# of recordings	%
Fitbit	[8, 24) hours	884	82.5
	[4, 8) hours	17	1.6
	[0, 4) hours	170	15.8
Atom	[4, 12) hours	418	87.3
	[2, 4) hours	22	4.6
	[0, 2) hours	39	8.1

This table shows the number of recordings according to their length. Each recording corresponds to one day of data.

##### Owl-in-One, TIS Devices, and Eddystone Beacons

These devices were subjected to a controlled experiment to verify that they ran continuously and to validate the data collection pipeline up to the daily dumps (see section Data acquisition and Flow). In addition, battery life experiments were run in a worst case controlled scenario and a 15 days autonomy was observed (increased to about 3 weeks in a real life setting due to a low power mode).

##### Atom

Tables 8 and 10 presents the information regarding the total number of hours recorded through the Atom phones, and the number of participants from which these recordings were obtained. The compliance rate was 52% and over 85% of the Atom recordings were over 4 hours. The decrease in compliance rate between the first, second, and third weeks of the study was not significant (Table 9).

#### Survey Data

Most of the scales used in the baseline scale had *α* values that were above the threshold of 0.70, which is commonly accepted as “good,” and that were comparable to those reported in the literature (see Table 6)^45^. Although the *α* values for agreeableness and open-mindedness scales were below those found in the literature, they were still above 0.70. The sleep disturbance and sleep somnolence scales, however, were below the threshold of 0.70. This may be due to the relatively small number of items in these scales (i.e., 4 and 3 items respectively). The coworker trust scale in the end of week survey was reliable (*α* = 0.76).

Table 11 demonstrates the opt-in rate for the EMAs, the percentage of completed EMAs, and the total number of completed EMAs by each type (i.e., midday, end of day, end of week). Most participants (>90%) answered at least one of the midday survey and one of the end of day surveys, while 80% of subjects attempted at least one end of week survey. The compliance rates for those subjects who opt-in to answer the midday survey and the end of day survey were 77.2% and 69.7% respectively. The compliance rate for the end of week surveys was 77.2%.

**Table 11:** Survey participation and compliance rates.

Survey	Kind	Opt-in	Compliance rate
		(% and *n* participants)	( % and *n* completed surveys)
EMAs	Midday	95% (54)	77.2% (866)
	End of Day	95% (54)	69.7% (780)
	End of Week	81% (46)	77.2% (105)

Opt-in rate is the percentage of participants that responded to a certain survey type. Compliance is measured as the percentage of completed surveys.

On average, the midday survey took under half a minute to complete (*M* = 0.41 mins, *SD* = 1.74) to complete. The end of day survey took, on average, 3.5 minutes to complete (*M* = 3.49 mins, *SD* = 7.13 mins) and the end of week survey took 7 minutes to complete (*M* = 6.90 mins, *SD* = 12.46 mins).

### Works using the Data Set

There are currently no works published using this data set.

## Usage Notes

### Data Access

Due to privacy concerns, we request a signed *Data Usage Agreement* (DUA) to grant access to all data records. A user signing this DUA agrees not to de-anonymize the data, identify language content, or share the data with individuals who have not signed a DUA. The document and the form to submit the signed document can be found here: https://tiles-data.isi.edu/download_tiles_2019. Once validated, the user will receive an email with the information on how to download each data record. The same account may be used for both TILES-2018 and TILES-2019 data sets, but different DUAs will be requested to access each data set.

### Main Record

The main data record has a total size of 25GB. To be mindful of the use of resources, we ask users to download this record only once.

### Audio Record

The audio record has a total size of 151GB. We provide the raw features collected and also the foreground masks that were inferred.

### Reading the Files

All files were compressed *Comma Separated Values* (CSV) files (.csv .gz), except for the foreground predictions which were stored as NumPy (.npy) files^46^. We recommend directly reading the compressed files. All files can be easily read in Python and R (using the Pandas library in Python^47^, or data.table^48^ or tidyverse^49^ in R). Note that .csv.gz files can also be opened directly in LibreOffice Calc^50^ (free software alternative to Microsoft Excel) without decompression. If the files need to be decompressed, we recommend using the command line utility gzip.

## Data Records: Use Cases

This data set was developed to study the well-being and team functioning of residents using a multi-modal approach. We envision several use cases for this data set.

### Signal processing

There are several opportunities for data quality enrichment in time series and graphs over time, including time series of Fitbit data or audio features, and graph-structured data corresponding to interactions. Like the TILES-2018^16^ data set, there are opportunities for the processing of longitudinal survey information, as well as for finding general representations of behavioral multimodal data.

### Workplace interactions

Researchers can examine interactions between residents and other members of their team (i.e., other residents, attendings, fellows, nurses) using social network analyses. Interaction data can be studied in conjunction with physiological data and with observed job performance data (i.e., attending and peer evaluations).

### Medical training

Researchers can also explore physiological and behavioral differences between residents who have worked in the ICU previously (i.e., second and third year residents) and those who have not (i.e., first year residents). Researchers may also be interested in the effects of witnessing patient deaths. 

## Data Availability

The code is available at https://github.com/usc-sail/tiles-2019-dataset/.
